# Net Carbon Sink Potential of Porous Vegetated Concrete: A Life-Cycle Assessment

**DOI:** 10.3390/ma19112237

**Published:** 2026-05-25

**Authors:** Hongquan Ren, Lingling Lu, Bing Tang, Tianbin Li

**Affiliations:** 1 State Key Laboratory of Geohazard Prevention and Geoenvironment Protection, Chengdu University of Technology, Chengdu 610059, China; ren20057438@126.com (H.R.); lulingling@stu.cdut.edu.cn (L.L.); 2 Sichuan Chengneiyu Expressway Co., Ltd., Chengdu 610041, China

**Keywords:** porous vegetated concrete, life cycle assessment, carbon emissions, vegetation carbon sequestration

## Abstract

Porous vegetated concrete has been widely used in highway slope protection because it provides both engineering stability and ecological restoration benefits. However, its life-cycle carbon emissions and long-term carbon sequestration performance have not been systematically quantified within a unified evaluation framework. In this study, 1 m^3^ of porous vegetated concrete was adopted as the functional unit, and a life-cycle assessment framework integrating carbon emissions and carbon sequestration was established. The results show that the material production stage is the dominant source of life-cycle carbon emissions, with cement consumption being the primary controlling factor. Under the system boundary and carbon sequestration assumptions adopted in this study, cumulative carbon sequestration over a 50-year service period was estimated to be approximately 470–475 kgCO_2_eq. This exceeded the corresponding life-cycle carbon emissions of 73–124 kgCO_2_eq and resulted in a net carbon sink potential of approximately 351–397 kgCO_2_eq. Based on equal weighting of 28-day shear strength and material production-stage carbon emissions, the efficacy coefficient method identified M2 as the preferred mix proportion for balancing mechanical performance and low-carbon objectives within the selected evaluation framework. Monte Carlo simulation confirmed the statistical stability of the estimated mean carbon emissions during the material production stage. Sensitivity analysis further showed that cement-related emissions and the vegetation carbon sequestration factor were the two most influential parameters affecting life-cycle carbon performance. Overall, this study provides a quantitative basis for evaluating the net carbon sink potential of porous vegetated concrete and offers decision support for low-carbon mix design in highway slope ecological protection engineering.

## 1. Introduction

In recent decades, intensified human engineering activities have led to a continuous increase in global carbon emissions. This has exacerbated the greenhouse effect and contributed to the increasing frequency of extreme weather events. Consequently, carbon emission reduction has become a major concern for both the international community and academia [1,2]. To address the ecological and societal risks associated with global warming, many countries signed the Paris Agreement in 2015. The agreement aims to limit the increase in global average temperature to well below 2 °C above pre-industrial levels and to pursue efforts to limit the increase to 1.5 °C [3]. Against this background, China has proposed the strategic goals of carbon peaking and carbon neutrality, aiming to accelerate the green and low-carbon transformation of high-emission sectors, including energy, industry, transportation, and buildings [4,5].

Concrete is the most widely used construction material worldwide. Its production accounts for approximately 8% of global carbon emissions [6], making it a key target for carbon reduction in the infrastructure sector. In transportation infrastructure, conventional concrete slope protection structures generally provide good stability during the early service stage. However, as service time increases, material deterioration, cracking, and poor conditions for vegetation growth may gradually occur. These problems can further lead to slope erosion, instability, and rockfall hazards [7,8,9]. Therefore, developing low-carbon slope protection technologies that integrate engineering safety and ecological functions has become an important direction for the sustainable development of transportation infrastructure.

Porous vegetated concrete is a slope protection material that combines structural stability with ecological restoration. In recent years, it has been increasingly applied in highway engineering. This technology typically integrates deep anchoring with anchor bolts, surface restraint by protective netting, and sprayed vegetated concrete. Together, these measures rapidly seal the slope surface, reduce rainwater erosion and infiltration, and provide a stable substrate for vegetation growth. Consequently, the stability and ecological functions of the shallow slope layer can be effectively enhanced [9,10]. Previous studies have shown that vegetated concrete can meet the mechanical performance requirements of slope protection. It also exhibits good water retention capacity, plant anchorage stability, and overall ecological and economic benefits [11,12]. To further improve its ecological performance and low-carbon potential, researchers have optimized the material system by incorporating additives such as biochar and recycled aggregates. These modifications can enhance the ecological adaptability and environmental sustainability of the material [13,14].

With growing emphasis on green and low-carbon development in transportation infrastructure, the carbon emission characteristics of vegetated concrete have received increasing scholarly attention. Existing studies have mainly examined how low-carbon cementitious materials, industrial by-products, and recycled aggregates affect the carbon emissions of vegetated concrete. For example, Kim et al. [15] reported that blast furnace slag aggregates can effectively reduce the carbon emission intensity of vegetated concrete. Faiz et al. [16] further reduced material-related emissions by combining low-alkalinity sulfoaluminate cement with biochar. Using a life-cycle assessment (LCA) framework, Zhu et al. [17] incorporated vegetation carbon sequestration into the carbon accounting of recycled concrete ecological bricks and examined the effects of aggregate replacement ratio and transportation distance on life-cycle carbon emissions. Luo et al. [10] and Liao et al. [18] evaluated the life-cycle carbon emission characteristics of vegetated concrete in riverbank protection and slope protection, respectively. Despite these advances, several limitations remain. Existing studies have mainly focused on carbon emission accounting during the material production stage, while insufficient attention has been paid to carbon sequestration during the operation stage, particularly the long-term sequestration associated with vegetation growth. In addition, most studies have reduced emissions primarily through material optimization, without systematically considering the combined effects of material system optimization and vegetation carbon sequestration enhancement on the life-cycle carbon balance. Therefore, under the “dual carbon” goals, it is necessary to establish a life-cycle low-carbon evaluation framework for porous vegetated concrete that integrates carbon emissions and carbon sequestration.

In this study, porous vegetated concrete was selected as the research object, and 1 m^3^ of material was defined as the functional unit. A life-cycle carbon accounting framework was established to cover the material production, transportation, construction, operation, and demolition stages. Within this framework, vegetation carbon sequestration and carbonation-related CO_2_ uptake by porous vegetated concrete were integrated into a unified evaluation system. A four-factor orthogonal experimental design involving cement, wood fiber, fly ash, and foaming agent was adopted to investigate the effects of mix proportions on 28-day shear strength and carbon emissions during the material production stage. The efficacy coefficient method was then used to comprehensively evaluate mechanical and low-carbon performance and to identify the preferred mix proportion. In addition, Monte Carlo simulation and sensitivity analysis were performed to assess the uncertainty of the carbon accounting results and to identify the key parameters affecting life-cycle carbon emissions and net carbon sink potential. This study provides a quantitative basis for the low-carbon mix design of porous vegetated concrete, the evaluation of net carbon sink potential, and the engineering application of ecological slope protection systems.

## 2. Methodology

### 2.1. Model Boundary

**Figure 1:**
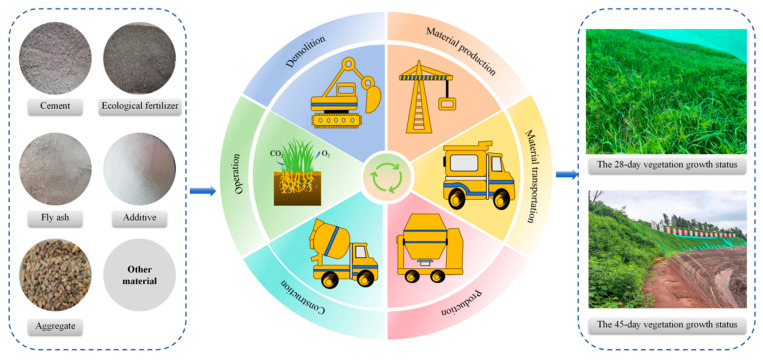
Model boundary.

This study employed life-cycle assessment (LCA) to systematically quantify the carbon emissions and vegetation carbon sequestration benefits of porous vegetated concrete throughout its life cycle, thereby evaluating its net carbon sink potential. The life-cycle carbon emissions were divided into six stages: material production, material transportation, porous vegetated concrete production, construction, operation, and demolition [17,19]. Because porous vegetated concrete is produced and sprayed directly on site, long-distance transportation of finished products is not involved. Therefore, a separate “finished product transportation” stage was not defined; the associated energy consumption was included in the construction stage for unified accounting. The system boundary is shown in Figure 1. To ensure comparability among different mix proportions, 1 m^3^ of porous vegetated concrete was defined as the functional unit, corresponding to a slope protection unit with a spraying thickness of 10 cm and a protected area of 10 m^2^. Within the system boundary adopted in this study, the operation stage includes carbonation-related CO_2_ uptake by porous vegetated concrete, carbon sequestration by vegetation growth, and carbon emissions from routine maintenance.

### 2.2. Calculation Methods for Carbon Emissions and Vegetation Carbon Sequestration

(1) Carbon emissions during the material production stage

Carbon emissions during the material production stage mainly arise from energy consumption associated with the extraction and processing of materials used in porous vegetated concrete. The calculation formula is given as follows:

(1) $$C_{1}=\sum_{i=1}^{n}M_{i}\times {EF}_{i}$$

In the formula, *C*_1_ denotes the carbon emissions from material production for 1 m^3^ of porous vegetated concrete; *M_i_* denotes the amount of the *i-th* material used per cubic meter; and *EF_i_* denotes the corresponding carbon emission factor.

(2) Carbon emissions during the material transportation stage

Carbon emissions during the material transportation stage mainly arise from fuel consumption by transportation vehicles. These emissions were calculated based on the transportation distance, material mass, and carbon emission factor per unit transport distance for the corresponding transportation mode. The calculation formula is given as follows:

(2) $$C_{2}=\sum_{i=1}^{n}M_{i}\times D_{i}\times T_{i}$$

In the formula, *C*_2_ denotes the carbon emissions during the material transportation stage; *D_i_* denotes the transportation distance of the *i-th* material; and *T_i_* denotes the carbon emission factor per unit mass-distance for the corresponding transportation mode. The transportation distance assumptions used in this stage are described in Section 3.3.

(3) Carbon emissions during the production stage of porous vegetated concrete

Carbon emissions during the production stage of porous vegetated concrete mainly arise from the energy consumption of machinery used for material mixing and related production processes. The calculation formula is given as follows:

(3) $$C_{3}=\sum_{j=1}^{m}E_{j}\times {EF}_{e,j}$$

In the formula, *C*_3_ denotes the carbon emissions during the production stage of porous vegetated concrete; *E_j_* denotes the energy consumption of the *j-th* type of machinery or equipment; and *EF*_*e*,*j*_ denotes the carbon emission factor corresponding to the j-th type of energy consumption.

(4) Carbon emissions during the construction stage of porous vegetated concrete

Carbon emissions during the construction stage mainly arise from the energy consumption of construction machinery and labor required for construction activities. According to the Highway Engineering Budget Quota (JTG/T 3832-2018) [20] and on-site construction practices, the main emission sources at this stage include equipment such as concrete spraying machines and air compressors, as well as labor required for construction activities. The calculation formula is given as follows:

(4) $$C_{4}=\sum_{i=1}^{k}E_{i}\times {EF}_{e,i}+L_{i}\times {EF}_{l}$$

In the formula, *C*_4_ denotes the carbon emissions during the construction stage; *E_i_* denotes the energy consumption of the *i-th* type of construction machinery; *EF*_*e*,i_ is the corresponding carbon emission factor of energy consumption; *L_i_* denotes the number of workers required for the *i-th* construction; and *EF_l_* denotes the carbon emission factor per unit of labor input.

(5) Carbon sequestration and carbon emission during the operation stage

The carbon balance during the operation stage mainly consists of three components: carbonation-related CO_2_ uptake by porous vegetated concrete, photosynthetic carbon sequestration by slope vegetation, and carbon emissions from routine maintenance. Based on field investigations at the project site and the Technical Guidelines for Ecological Protection of Highway Slopes with Wire Mesh, Anchors, and Sprayed Vegetated Concrete (DB 50/51/T 10006–2023) [21], routine maintenance of porous vegetated concrete ecological slope protection is mainly concentrated in the early stage of vegetation establishment. Therefore, the operation-stage carbon balance was evaluated under a stable vegetation growth scenario. Under this scenario, slope vegetation gradually forms a relatively stable cover after establishment and maintains stable growth during the subsequent operation period. The calculation formulas are given as follows:

(5) $$C_{5}=C_{5a}+C_{5b}$$

The carbonation-related CO_2_ uptake of porous vegetated concrete, *C*_5*a*_, was estimated based on the CO_2_ absorption ratio for pervious concrete reported by Ellingboe et al. [22]:

(6) $$C_{5a}=(C_{1}+C_{2}+C_{3})\times 8\%$$

(7) $$C_{5b}=A_{s}\times S_{s}\times T$$

(8) $$C_{5c}=N(\sum_{i=1}^{k}M_{m,i}\times {EF}_{i}+E_{m,i}\times {EF}_{e,i}+L_{m}\times {EF}_{l})$$

In the formulas, *C*_5_ denotes the carbon sequestration of the porous vegetated concrete during the operation stage; *C*_5*a*_ denotes the carbonation-related CO_2_ uptake of porous vegetated concrete through carbonation; *C*_5*b*_ denotes carbon sequestration by vegetation during the operation stage; and *C*_5*c*_ denotes carbon emissions from routine maintenance during the operation stage. *As* is the annual carbon sequestration factor per unit area of vegetation, *Ss* is the vegetation-covered area, and *T* is the service life. *N* denotes the number of maintenance activities during the operation stage. *M*_*m*,*i*_ denotes the amount of the *i-th* material consumed in a single maintenance activity, and *EF_i_* denotes the corresponding carbon emission factor. *E*_*m*,*i*_ is the energy consumed by the i-th type of machinery in a single maintenance activity, and *EF*_*e*,*i*_ denotes the carbon emission factor of the corresponding energy source. *L_m_* denotes the labor input required for a single maintenance activity, and *EF_l_* denotes the carbon emission factor per unit of labor input.

(6) Carbon emissions during the demolition stage

Carbon emissions during the demolition stage of porous vegetated concrete mainly arise from the energy consumption of demolition machinery. Based on previous studies and by analogy with previous concrete demolition, emissions at this stage were estimated as 90% of the carbon emissions generated during the construction stage [17,20]. The calculation formula is given as follows:

(9) $$C_{6}=C_{4}\times 0.9$$

In the formula, *C*_6_ denotes the carbon emissions during the demolition stage of porous vegetated concrete.

### 2.3. Efficacy Coefficient Method

The efficacy coefficient method is a multi-attribute decision-making approach that assigns a quantitative coefficient to each evaluation indicator. It is suitable for decision-making problems involving multiple factors. The basic principle is to convert each original indicator value into a dimensionless efficacy coefficient, which reflects its relative contribution to the overall performance. In multi-indicator evaluations, different indicators often have different dimensions and levels of influence on comprehensive performance [23]. Therefore, the efficacy coefficient method was adopted in this study to evaluate the mix proportions of porous vegetated concrete. The original indicators were first normalized into dimensionless efficacy values, and then a weighted comprehensive efficacy coefficient was calculated to support unified comparison and ranking among different mix proportions [24]. The normalization procedures are given in Equations (10) and (11).

Benefit-oriented indicators:

(10) $$d_{ji}=\frac{C_{ji}}{C_{\max}}$$

Cost-oriented indicators:

(11) $$d_{ji}=\frac{C_{\min}}{C_{ji}}$$

Based on the efficacy coefficients calculated for each evaluation indicator, the comprehensive efficacy coefficient *D_j_* of the *j-th* mix proportion was determined using the weighted geometric mean method, as shown in Equations (12) and (13):

(12) $$D_{j}=\prod_{i=1}^{m}d_{ji}^{wi}$$

(13) $$\sum_{i=1}^{m}w_{i}=1$$

In Equations (12) and (13), *D_j_* denotes the comprehensive efficacy coefficient of the *j-th* mix proportion; *d_ji_* denotes the efficacy coefficient of the *j-th* mix proportion for the *i-th* evaluation indicator; and *w_i_* denotes the weight assigned to the *i-th* evaluation indicator. In this study, 28-day shear strength and carbon emissions during the material production stage were selected as the two evaluation indicators. Equal weights were assigned to these two indicators, namely w_shear_ = w_CO2_ = 0.5.

Therefore, for the two equally weighted indicators, the comprehensive efficacy coefficient can be expressed as shown in Equation (14):

(14) $$D_{j}=d_{j,shear}^{0.5}\times d_{j,{CO}_{2}}^{0.5}$$

### 2.4. Uncertainty Analysis of the Carbon Emission Model

Given that most carbon emission factors reported in standards and previous studies are provided as recommended values, sufficient data are often unavailable to define their probability distributions rigorously. Therefore, Monte Carlo simulation was used to evaluate the uncertainty of carbon emissions during the material production stage [1,2]. In this analysis, the carbon emission factors of key materials were treated as normally distributed random variables. The nominal value of each factor was used as the mean, and the standard deviation was set to 10% of the nominal value [1,2]. A total of 10,000 random samples were generated, and the corresponding carbon emissions were calculated. The output distributions were then statistically analyzed to assess the robustness of the estimated mean carbon emissions during the material production stage [2].

## 3. Mix Proportion Design and Key Parameters

### 3.1. Material Proportioning of Porous Vegetated Concrete

To satisfy the dual objectives of engineering performance and low-carbon design in slope ecological protection, an L_16_(4^4^) orthogonal experimental design with four factors and four levels was employed to optimize the mix proportions of porous vegetated concrete. This design enables efficient testing and statistical comparison using a limited number of experimental groups. The factor levels were selected based on previous studies by our research group [25,26,27] and the local technical standard, Technical Guidelines for Ecological Protection of Highway Slopes with Net Anchor Shotcrete Vegetated Concrete (DB 50/51/T 10006–2023) [21]. The four key factors considered were cement, wood fiber, fly ash, and foaming agent.

(1) Cement:

Cement serves as the primary binder in porous vegetated concrete, and its dosage directly affects the strength and erosion resistance of the material. An appropriate cement content enhances structural stability, whereas excessive cement use substantially increases carbon emissions. P.O 42.5 ordinary Portland cement was used in this study. Accordingly, the cement dosage levels were set at 30, 50, 70, and 90 kg/m^3^.

(2) Wood fiber:

Wood fiber improves the pore structure and water retention capacity of porous vegetated concrete, thereby providing a favorable microenvironment for vegetation growth. However, excessive wood fiber content may reduce mechanical strength and overall stability. Accordingly, the wood fiber dosage levels were set at 15, 20, 25, and 30 kg/m^3^.

(3) Fly ash:

Fly ash, a commonly used mineral admixture, improves the workability and durability of the mixture and can partially replace cement, thereby reducing carbon emissions during material production. Accordingly, the fly ash content levels were set at 20, 40, 60, and 80 kg/m^3^.

(4) Foaming agent:

An appropriate amount of foaming agent promotes the formation of a connected pore structure, improving both drainage and water retention. However, excessive foaming agent content may reduce shear strength, increase porosity, and accelerate water loss, which are unfavorable for seed germination and seedling growth. Accordingly, the foaming agent dosage levels were set at volume ratios of 20%, 25%, 30%, and 35%.

In addition to the four variables in the orthogonal experiment, the other components were kept constant across all mix proportions, including planting soil, aggregate, additives, ecological fertilizer, and water. The planting soil was red-bed clay from the Sichuan Basin, and the aggregate consisted of crushed stone and fine sand. The additives mainly included a binder, a water-retaining agent, and a pH-regulating agent. The ecological fertilizer contained magnetic mineral materials and was used to provide a sustained nutrient supply for vegetation growth. The orthogonal experimental design is shown in Table 1.

### 3.2. Experimental Methods

Direct shear tests were conducted using a DZJ-4 multifunctional portable direct shear apparatus. Specimen preparation and testing procedures followed the Standard for Geotechnical Testing Methods (GB/T 50123-2019) [28]. The specimen ring had a diameter of 6.18 cm and a height of 2.0 cm. All specimens were cured under identical conditions for 28 days before testing. During the tests, normal stresses of 50, 100, 150, and 200 kPa were applied. The shear rate was controlled at 0.8 mm/min, and each test was terminated when the shear displacement reached 10 mm.

### 3.3. Carbon Emissions and Carbon Sequestration Factors

To ensure the scientific validity and comparability of the life-cycle carbon accounting results, carbon emission and carbon sequestration factors were selected according to the principles of “local priority, authoritative traceability, and data timeliness”. Domestic databases and standards, including the China Product Life Cycle Greenhouse Gas Emission Coefficient Database [29] and the Building Carbon Emission Calculation Standard (GB/T 51366-2019) [30], were preferentially adopted and supplemented by relevant literature. For parameters without directly matched database entries, such as the foaming agent, additives, ecological fertilizer, and fly ash, the factors were determined based on relevant literature, material characteristics, and engineering practice. The selected factors and their data sources are summarized in Table 2 and Table 3.

## 4. Results and Discussion

### 4.1. Shear Strength and Carbon Emission Characteristics During Material Production Stage

The results of the orthogonal experiment are presented in Table 4. The table summarizes the 28-day shear strength and material production-stage carbon emissions of the 16 porous vegetated concrete mix proportions designed according to the orthogonal scheme shown in Table 1. To ensure consistent evaluation of shear performance among different mix proportions, the peak shear stress obtained from the direct shear tests under a normal stress of 150 kPa was adopted as the 28-day shear strength indicator. For each mix proportion, three replicate tests were conducted, and the mean shear strength and corresponding standard deviation are reported in Table 4. The mean shear strength was then used as the mechanical performance indicator in the subsequent efficacy coefficient method. To further evaluate whether the differences in shear strength among the mix proportions were statistically significant, a one-way analysis of variance was performed using the replicate test results of the 16 mix proportions. The results showed that the 28-day shear strength differed significantly among the mix proportions (*p* < 0.05). This indicates that changes in the overall mix proportion had a significant effect on the shear strength of porous vegetated concrete.

**Figure 2:**
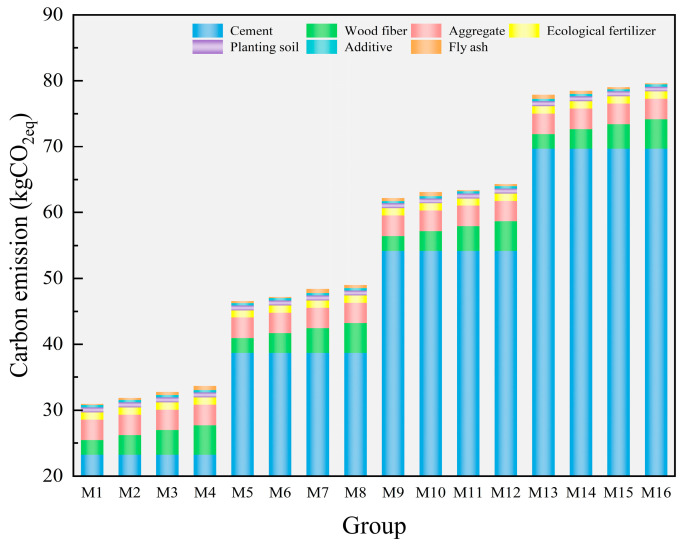
Carbon emissions during the material production stage.

Figure 2 illustrates the carbon emissions during the material production stage. The results show a clear stepwise distribution of carbon emissions among different porous vegetated concrete mix proportions, indicating that mix proportion design has a substantial influence on material production-stage emissions. This variation is mainly attributed to differences in the dosages of key materials. The individual carbon emissions from water and foaming agent are each less than 0.5 kgCO_2_eq and therefore make negligible contributions to the total emissions; consequently, they are not shown separately in Figure 2. The itemized carbon emissions of the main materials for each mix proportion are provided in Supplementary Table S1 as a quantitative supplement to Figure 2.

From the perspective of carbon emission composition, cement consistently dominated carbon emissions during the material production stage, and its contribution increased markedly with increasing cement content. In the low-emission group (M1–M4), cement emissions reached 23.22 kgCO_2_eq, accounting for approximately 70% of total material production-stage emissions. In the high-emission group (M13–M16), cement emissions increased to 69.66 kgCO_2_eq, with the contribution rising to approximately 89%. This trend indicates that cement became increasingly dominant in material production-stage emissions as its dosage increased. Under high-cement conditions, the high carbon intensity of cement gradually outweighed the emission reduction benefits provided by low-carbon Supplementary Materials such as fly ash. Although Supplementary Materials still contributed to emission reduction, their relative effect was weakened as cement content increased. Therefore, controlling the absolute cement dosage is more fundamental for low-carbon optimization than relying solely on partial replacement with Supplementary Materials. These findings are consistent with life-cycle assessment results reported for recycled fine aggregate concrete and hydroseeded concrete, in which cement was identified as the primary contributor to material production-stage carbon emissions [10,33]. By comparison, wood fiber contributed relatively little to total emissions across all mix proportions, with emissions ranging from 2.2 to 4.5 kgCO_2_eq. Baltrocchi et al. [34] also showed that recycled bio-based fibers can help reduce the environmental burdens of construction material systems. Carbon emissions from aggregates and ecological fertilizer remained relatively stable, at approximately 3.1 kgCO_2_eq and 1.1 kgCO_2_eq, respectively. Other materials, such as planting soil and fly ash, contributed only marginally to total emissions during the material production stage. Overall, effective control of cement content and systematic optimization of material composition are key strategies for reducing material production-stage carbon emissions.

**Figure 3:**
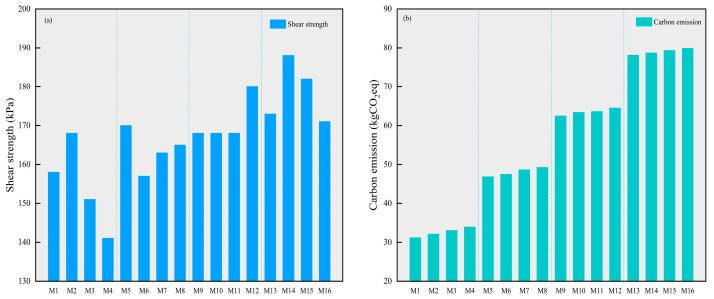
Relationship between shear strength and carbon emissions during material production. (**a**) 28-day shear strength; (**b**) Carbon emissions during the material production stage.

The above analysis indicates that carbon emissions during the material production stage show distinct grouping characteristics under different mix proportions, and this variation is mainly governed by cement content. To further examine the effects of mix proportions on mechanical performance and carbon emissions, 28-day shear strength and material production-stage carbon emissions were selected as key indicators for comparative analysis, as shown in Figure 3.

Carbon emissions during the material production stage showed a distinct grouping pattern. Specifically, M1–M4 formed the low-carbon emission group (31–34 kgCO_2_eq), M5–M8 formed the medium-carbon emission group (46–50 kgCO_2_eq), M9–M12 formed the relatively high-carbon emission group (62–65 kgCO_2_eq), and M13–M16 formed the high-carbon emission group (78–79 kgCO_2_eq). This stepwise trend indicates that material production-stage carbon emissions were mainly governed by cement content and increased approximately linearly with increasing cement dosage. It further confirms that cement, as the primary binder, was the dominant source of carbon emissions at this stage, and that variations in cement dosage largely determined the emission level of each mix proportion.

To further quantify the relative influence of each material factor, an orthogonal range analysis was conducted using 28-day shear strength and material production-stage carbon emissions as evaluation indicators. The results are presented in Table 5. For material production-stage carbon emissions, cement showed the largest range value of 46.44 kgCO_2_eq, which was much higher than those of wood fiber, fly ash, and foaming agent, with values of 2.25, 0.48, and 0.09 kgCO_2_eq, respectively. This result confirms that differences in carbon emissions among the mix proportions were mainly controlled by cement dosage. By contrast, 28-day shear strength was influenced by multiple material factors. Cement showed the largest range value of 24.00 kPa, followed by fly ash, foaming agent, and wood fiber, with range values of 13.75, 10.25, and 6.00 kPa, respectively. These results indicate that material production-stage carbon emissions were primarily governed by cement content, whereas shear strength was jointly affected by cement, fly ash, foaming agent, and wood fiber.

The range analysis also helps explain the positive relationship between shear strength and carbon emissions shown in Figure 3. Overall, shear strength increased with increasing carbon emissions. Specifically, the shear strength ranged from 141 to 168 kPa in the low-carbon emission group, 157–170 kPa in the medium-carbon emission group, 168–180 kPa in the relatively high-carbon emission group, and 171–188 kPa in the high-carbon emission group. This relationship can be mainly attributed to the role of cement. As the primary binder, cement improves the integrity of the structural skeleton and the interfacial bonding performance of porous vegetated concrete. At the same time, it is also the dominant source of carbon emissions during the material production stage. Therefore, increasing cement content tends to enhance shear strength while also increasing carbon emissions. These findings are consistent with previous studies. Faiz et al. [16] reported that optimizing the proportions of sulfoaluminate cement and biochar in biochar-based vegetated concrete systems can improve engineering performance while reducing carbon emissions and improving the plant growth environment. Similarly, Wang et al. [35] showed through orthogonal experiments on lithium slag recycled planting concrete that an appropriate reduction in cement content can provide environmental benefits. Therefore, in practical engineering applications, the sustainable optimization of porous vegetated concrete should focus on mix design optimization and the balance between mechanical performance and environmental benefits, while ensuring sufficient safety margins and durability requirements.

### 4.2. Life-Cycle Carbon Emission Characteristics and Net Carbon Sink Potential

**Figure 4:**
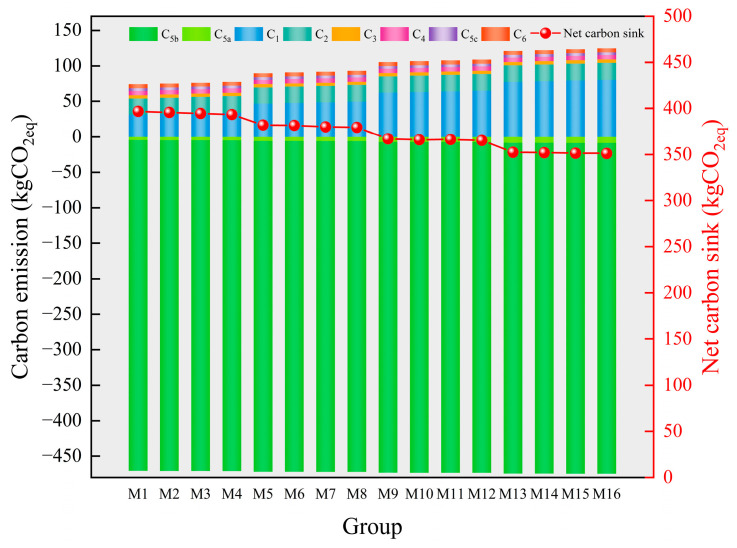
Life-cycle carbon emissions and net carbon sink characteristics of porous vegetated concrete.

To clarify the effects of different orthogonal mix proportion schemes on the overall carbon performance of porous vegetated concrete, this section evaluates the life-cycle carbon emissions, carbon sequestration, and net carbon sink characteristics of each scheme. The evaluation is based on the experimental design described in Section 3 and the calculation method presented in Section 2.2. The analysis covers the material production, transportation, porous vegetated concrete production, construction, operation, and demolition stages. It provides an integrated assessment of stage-wise carbon contributions and life-cycle carbon performance under different mix proportion conditions. The calculation results are shown in Figure 4.

Figure 4 shows the life-cycle carbon emissions, carbon sequestration, and net carbon sink characteristics of porous vegetated concrete under different mix proportions. Overall, life-cycle carbon emissions increased stepwise with increasing material dosage, indicating that mix proportion design strongly affects the carbon performance of porous vegetated concrete. In terms of stage-wise contributions, the material production stage was consistently the dominant source of life-cycle carbon emissions, accounting for 42–65% of the total emissions. This result is consistent with the findings of Kim et al. [15] for porous vegetated concrete and Zhu et al. [17] for recycled aggregate concrete, both of which identified material production as the main contributor to carbon emissions. By contrast, Lei et al. [33] reported that the material production stage accounted for 80.92–85.62% of total carbon emissions in recycled fine aggregate concrete, which is higher than the values obtained in this study. This difference suggests that optimized mix proportion design may reduce the relative contribution of the material production stage to total life-cycle carbon emissions and thereby improve overall environmental performance.

As material dosages increased, total life-cycle carbon emissions showed a trend consistent with the carbon emissions from the material production stage. This further indicates that differences in the consumption of key materials were the main driver of variations in total life-cycle carbon emissions. By contrast, carbon emissions from the transportation, production, construction, and demolition stages remained relatively stable across different mix proportions. Therefore, these stages had only a limited effect on the overall life-cycle carbon balance. This observation is generally consistent with previous studies [17,33,36]. During the operation stage, the carbon sequestration amounts of different mix proportions showed only minor differences. This is because the same vegetation-covered area and annual carbon sequestration factor per unit area were adopted for all schemes; therefore, the vegetation carbon sequestration amount was identical. The differences in carbon sequestration among the mix proportions mainly resulted from variations in carbonation-related CO_2_ uptake by porous vegetated concrete. When both vegetation carbon sequestration and carbonation-related CO_2_ uptake by porous vegetated concrete were considered, the cumulative carbon sequestration amounts under the 25-year and 50-year scenarios were approximately 237–242 kgCO_2_eq and 470–475 kgCO_2_eq, respectively. The corresponding net carbon sinks were approximately 118–163 kgCO_2_eq and 351–397 kgCO_2_eq, respectively. These results indicate that porous vegetated concrete can maintain a positive net carbon balance within the accounting boundary and carbon sequestration assumptions adopted in this study. However, the magnitude of the net carbon sink is sensitive to the assumed service life. This finding is consistent with previous studies, which reported that vegetation can continuously provide carbon sequestration through photosynthesis during long-term operation [2,10].

In terms of variation trends, although carbon sequestration increases slightly with increasing mix proportion levels during the operation stage, the net carbon sink generally declines because material-related carbon emissions increase more substantially. This finding indicates that the environmental performance of porous vegetated concrete depends not only on the long-term carbon sequestration capacity of vegetation but also on the effective control of carbon emissions during the material production stage. Therefore, considering the dominant contribution of the material production stage to total life-cycle carbon emissions and the engineering importance of mechanical performance, the subsequent optimization focuses on balancing material production-stage carbon emissions and shear strength within a unified low-carbon evaluation framework.

### 4.3. Determination of the Optimal Mix Proportion Using the Efficacy Coefficient Method

**Figure 5:**
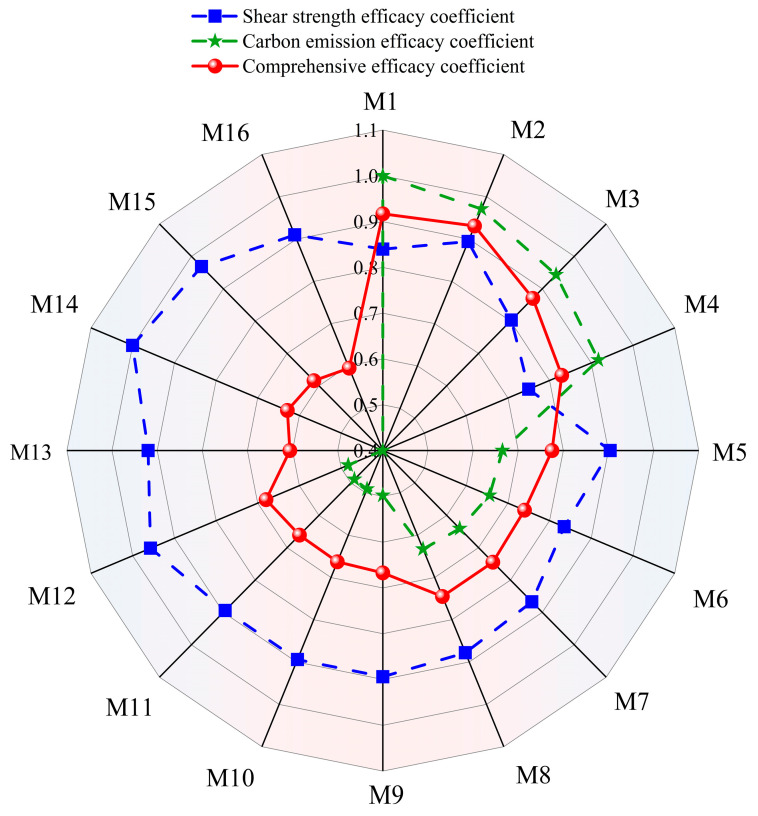
Comparison of the comprehensive efficacy coefficients of different mix proportions.

The above analysis indicates that the material production stage is the main source of carbon emissions throughout the life cycle, while shear strength is a key indicator for evaluating the engineering performance of porous vegetated concrete. Based on this, shear strength and material production-stage carbon emissions were selected as the core evaluation indicators in this study. The efficacy coefficient method was then applied for multi-objective comprehensive assessment to determine the optimal factor-level combination and the preferred mix proportion. The comprehensive assessment results of different mix proportions are shown in Figure 5, and the detailed calculation results are provided in Supplementary Table S2. The results indicate a clear trade-off between shear strength and carbon emissions among different mix proportions.

Among the design schemes, group M2 showed the highest comprehensive efficacy coefficient of 0.931, indicating the best overall performance. This result suggests that M2 effectively reduced carbon emissions while maintaining high shear strength, thereby achieving an optimal balance between mechanical performance and low-carbon objectives. Groups M1 and M3 also showed strong overall performance, with comprehensive efficacy coefficients of 0.917 and 0.870, respectively. This reflects a favorable synergy between low-carbon characteristics and mechanical performance and indicates their potential for engineering application. By contrast, groups M13–M16 generally exhibited lower comprehensive efficacy coefficients, mainly because their substantially higher carbon emissions weakened their overall performance. Although some schemes did not achieve the highest value for a single mechanical indicator, they still obtained relatively high comprehensive efficacy coefficients because of their moderate carbon emission levels. Therefore, the efficacy coefficient method can effectively integrate multidimensional indicators, such as mechanical performance and environmental impact. It also reveals the relative advantages and disadvantages of different mix proportions under multi-objective constraints, providing a scientific basis for optimizing ecological material proportions and selecting engineering schemes under low-carbon requirements.

### 4.4. Uncertainty and Sensitivity Analysis

**Figure 6:**
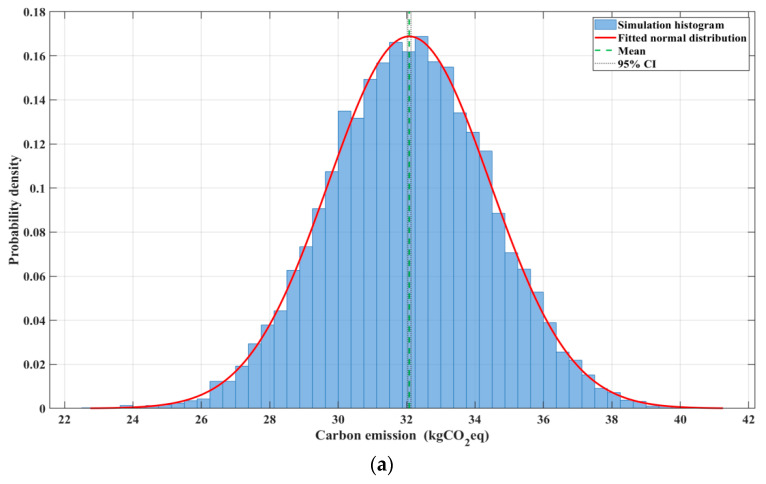

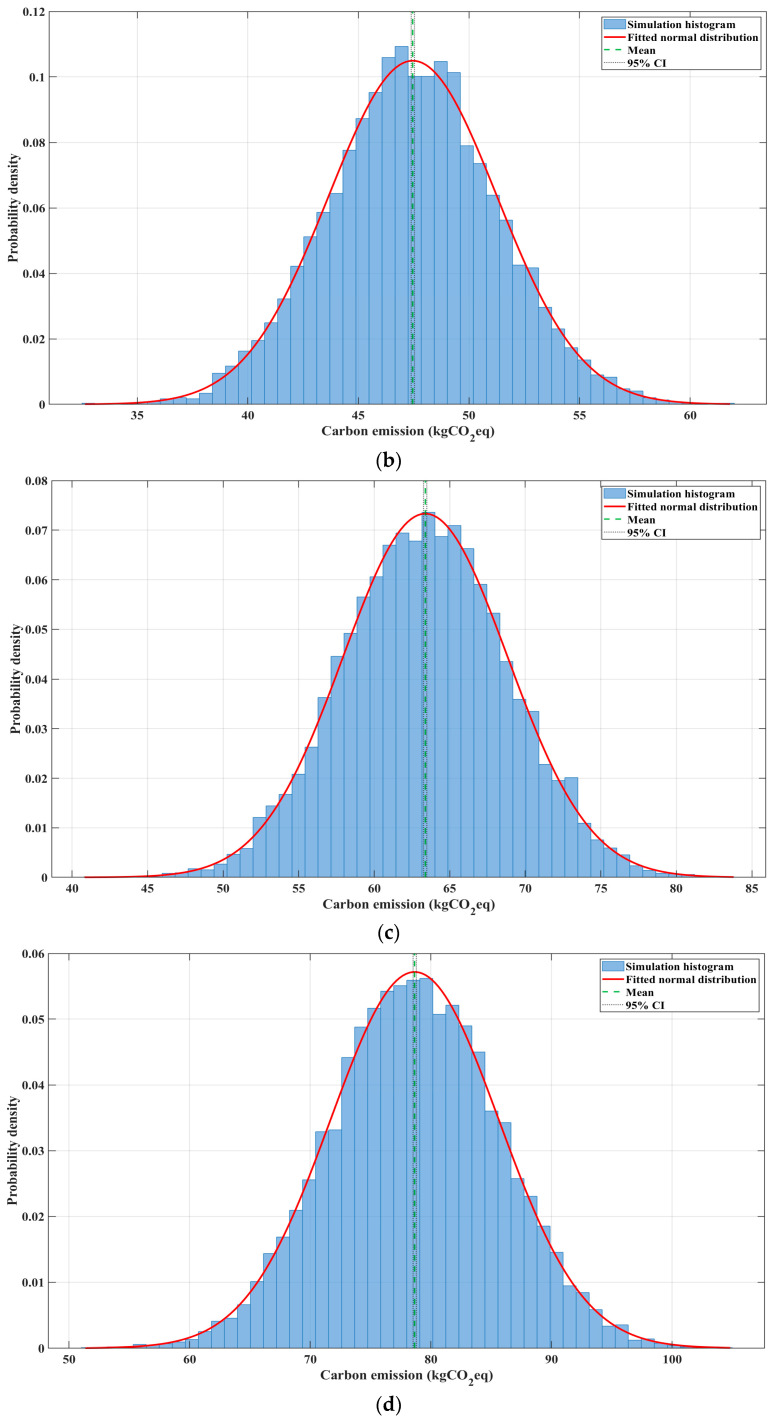
Probability distributions of carbon emissions during the material production stage for typical mix proportion schemes based on 10,000 Monte Carlo simulations. (**a**) Monte Carlo distribution for mix proportion M2; (**b**) Monte Carlo distribution for mix proportion M6; (**c**) Monte Carlo distribution for mix proportion M10; (**d**) Monte Carlo distribution for mix proportion M14.

Given that the material production stage dominates the carbon emissions of porous vegetated concrete throughout its life cycle, with a substantially higher contribution than the transportation, construction, and demolition stages, and considering the wide variability and significant differences in carbon emission factors at this stage, it represents the primary source of uncertainty in the life cycle assessment. To ensure the representativeness and typicality of the analysis, four representative mix proportions were selected for uncertainty analysis: the low-carbon group M2, the medium-carbon group M6, the relatively high-carbon group M10, and the high-carbon group M14. Table 6 summarizes the statistical results of carbon emissions during the material production stage, and Figure 6 shows the corresponding probability density distributions and normal fitting results obtained from the Monte Carlo simulations.

As shown in Table 6, the mean carbon emissions increase progressively from M2 to M14. The average emissions for M2, M6, M10, and M14 are 32.08, 47.46, 63.39, and 78.65 kgCO_2_eq, respectively, with 95% confidence intervals of [32.03, 32.13], [47.39, 47.53], [63.29, 63.50], and [78.51, 78.79]. The relatively narrow 95% confidence intervals indicate that the estimated mean values obtained from 10,000 Monte Carlo simulations are statistically stable. As shown in Figure 6, the carbon emission distributions for each mix proportion are approximately normal, and the fitted curves exhibit smooth distribution patterns. This suggests that the Monte Carlo sampling size is sufficient to provide robust estimates of the mean carbon emissions during the material production stage [1,2].

As shown in Table 6 and Figure 6, the estimated carbon emissions during the material production stage showed good overall statistical stability. However, differences among the representative mix proportions remained evident. This indicates the need to identify the dominant sources of variability and quantify the influence of key parameters on life-cycle carbon emissions and carbon sequestration. Therefore, a sensitivity analysis was further conducted. The analysis focused on the carbon emission factors of cement, wood fiber, and aggregate, as well as the vegetation carbon sequestration factor per unit area. The four representative mix proportions were used as case studies. Fly ash and foaming agent were not included as key sensitivity parameters because their contributions to total life-cycle carbon emissions were relatively small under the investigated mix designs. The sensitivity of each parameter was examined using perturbation levels of ±5%, ±10%, and ±15% [10].

**Figure 7:**
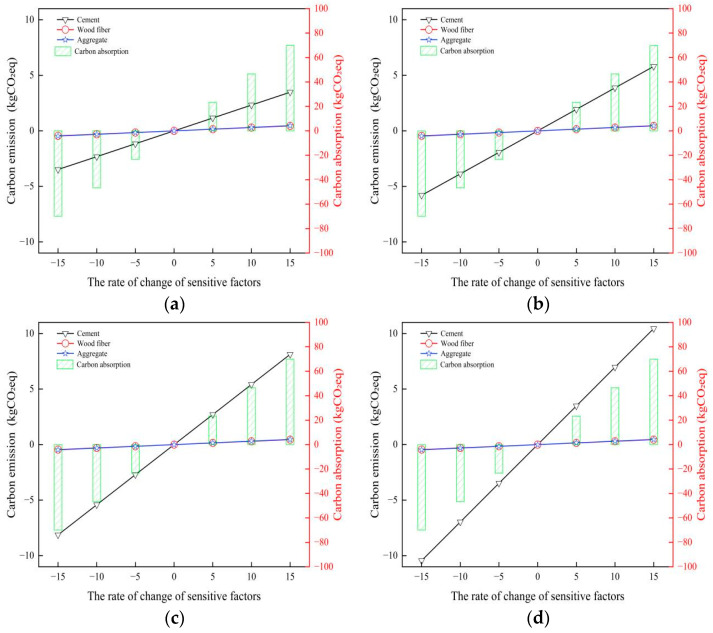
Sensitivity analysis of typical mix proportion schemes. (**a**) Sensitivity analysis of mix proportion M2; (**b**) Sensitivity analysis of mix proportion M6; (**c**) Sensitivity analysis of mix proportion M10; (**d**) Sensitivity analysis of mix proportion M14.

As shown in Figure 7, the cement carbon emission factor was consistently the most influential parameter affecting life-cycle carbon emissions, and its sensitivity increased with cement dosage. When this factor was perturbed by ±15%, the carbon emissions of M2, M6, M10, and M14 changed by ±3.48, ±5.80, ±8.13, and ±10.45 kgCO_2_eq, respectively. By contrast, the effects of the wood fiber and aggregate carbon emission factors were relatively small, resulting in changes of approximately ±0.45 kg CO_2_eq and ±0.47 kgCO_2_eq, respectively. The vegetation carbon sequestration factor per unit area also showed high sensitivity during the long-term operation stage. In this study, the annual vegetation carbon sequestration factor was set to 0.932 kgCO_2_eq/(m^2^·a) [32]. Considering that vegetation carbon sequestration may vary with plant community composition, growth stage, climatic conditions, substrate environment, and maintenance status, a ±15% variation was applied in the sensitivity analysis. Under this variation, vegetation carbon sequestration changed by approximately ±69.9 kgCO_2_eq per 10 m^2^ over a 50-year service life, indicating that the estimated net carbon sink is sensitive to this parameter. In addition, the net carbon sink estimation relies on the assumption that porous vegetated concrete and slope vegetation maintain stable performance over the 50-year service life. Due to the high porosity of porous vegetated concrete, long-term durability factors, such as carbonation progression, freeze–thaw cycles, corrosive ion migration, and wet–dry cycles, may affect matrix stability and sustained vegetation growth. Accordingly, the reported net carbon sink represents a scenario-based estimate under the defined material boundary, service-life assumption, and carbon sequestration parameters. Therefore, plant species selection, community stability, and substrate optimization should be considered in engineering applications to enhance the long-term carbon sequestration potential of porous vegetated concrete systems.

Therefore, to achieve life-cycle low-carbon optimization of porous vegetated concrete, a synergistic strategy combining emission reduction and carbon sequestration enhancement should be adopted. On the emission reduction side, priority should be given to reducing the carbon intensity of cement by optimizing the mix proportion, lowering cement consumption, using low-carbon cement substitutes, and improving production processes and energy structures [36]. On the carbon sequestration side, vegetation carbon sequestration should be enhanced by selecting plant species with high carbon sequestration efficiency and strong environmental adaptability. Combined with substrate improvement and appropriate maintenance management, this strategy can support long-term biomass accumulation and sustained carbon storage [2].

## 5. Conclusions

This study establishes an integrated evaluation framework that jointly considers life-cycle carbon emissions and long-term vegetation carbon sequestration. Using this framework, the net carbon sink potential of porous vegetated concrete is quantified, and support is provided for low-carbon mix optimization in slope ecological protection. The main conclusions are as follows.

(1) Under the system boundary and carbon sequestration assumptions adopted in this study, porous vegetated concrete exhibited a certain net carbon sink potential at the life-cycle scale. Over a 50-year service period, cumulative carbon sequestration was estimated to be approximately 470–475 kgCO_2_eq, exceeding the total life-cycle carbon emissions of 73–124kg CO_2_eq. Accordingly, the resulting net carbon sink was approximately 351–397kg CO_2_eq. These results indicate that porous vegetated concrete can provide slope protection and ecological restoration benefits while also contributing to a measurable net carbon sink within the defined accounting boundary.
(2) The material production stage dominates life-cycle carbon emissions, accounting for 42–65% of the total. Cement is the primary controlling factor at this stage, with its contribution increasing from approximately 70% in the low-carbon group to 89% in the high-carbon group. This finding indicates that controlling cement dosage is the most effective approach for reducing material-related carbon emissions.
(3) Carbon emissions during the material production stage showed a clear positive correlation with the 28-day shear strength, indicating a trade-off between mechanical performance and low-carbon objectives. Based on the efficacy coefficient method, M2 was identified as the optimal mix design. This mixture, consisting of 30 kg/m^3^ cement, 20 kg/m^3^ wood fiber, 40 kg/m^3^ fly ash, and 25% foaming agent, achieved the highest efficacy coefficient of 0.931.
(4) Monte Carlo simulation confirmed the statistical stability of the carbon emission estimates for the material production stage. The mean carbon emissions of M2, M6, M10, and M14 were 32.08, 47.46, 63.39, and 78.65 kgCO_2_eq, respectively, with relatively narrow 95% confidence intervals. These results demonstrate the robustness of the estimated mean carbon emissions. Sensitivity analysis further indicated that the cement emission factor was the dominant contributor to life-cycle carbon emissions, whereas the vegetation carbon sequestration factor had a substantial influence on the long-term net carbon sink. Therefore, future low-carbon design of porous vegetated concrete should focus on reducing cement-related emissions while enhancing vegetation carbon sequestration capacity, thereby improving long-term net carbon sink performance.

This study still has several limitations. The vegetation carbon sequestration factor was derived from literature-based annual average values and may not fully reflect differences in plant species, growth stages, or local environmental conditions. In addition, this study assumes a relatively stable carbon sequestration rate over the 50-year service period and does not explicitly account for vegetation succession, species replacement, or extreme climate events. Future research should integrate long-term field monitoring, native plant configurations, and dynamic carbon-balance modeling to improve the accuracy of net carbon sink assessment for porous vegetated concrete.

## Figures and Tables

**Table 1:** Orthogonal experimental design scheme.

Group	Cement (kg/m^3^)	Wood Fiber (kg/m^3^)	Fly Ash (kg/m^3^)	Foaming Agent %	Planting Soil (kg/m^3^)	Aggregate (kg/m^3^)	Additive (kg/m^3^)	Ecological Fertilizer (kg/m^3^)	Water (kg/m^3^)
M1	30	15	20	20	1500	400	18	15	300
M2	30	20	40	25	1500	400	18	15	300
M3	30	25	60	30	1500	400	18	15	300
M4	30	30	80	35	1500	400	18	15	300
M5	50	15	40	30	1500	400	18	15	300
M6	50	20	20	35	1500	400	18	15	300
M7	50	25	80	20	1500	400	18	15	300
M8	50	30	60	25	1500	400	18	15	300
M9	70	15	60	35	1500	400	18	15	300
M10	70	20	80	30	1500	400	18	15	300
M11	70	25	20	25	1500	400	18	15	300
M12	70	30	40	20	1500	400	18	15	300
M13	90	15	80	25	1500	400	18	15	300
M14	90	20	60	20	1500	400	18	15	300
M15	90	25	40	35	1500	400	18	15	300
M16	90	30	20	30	1500	400	18	15	300

**Table 2:** Carbon Emission Factors for Materials, Energy, and Construction Activities.

Item	Unit	Carbon Emission Factor (kgCO_2_eq/Unit)	References
Cement	kg	0.774	CPCD [29]
Wood fiber	kg	0.15	CPCD [29]
Fly ash	kg	0.008	Sathiparan et al. [31]
Foaming agent	m^3^	0.638	--
Planting soil	kg	0.0005	Xiao et al. [32]
Aggregate	kg	0.00776	CPCD [29]
Additive	kg	0.02	--
Ecological fertilizer	kg	0.0742	--
Water	kg	0.000168	(GB/T 51366-2019) [30]
Diesel oil	kg	3.16	Xiao et al. [32]
Gasoline	kg	2.98	Xiao et al. [32]
Electric power	kW·h	0.58	Xiao et al. [32]
Labor	d	1.024	Luo et al. [10]
Light gasoline truck transportation	t·km	0.334	(GB/T 51366-2019) [30]

Note 1: In Table 2, Note: The unit “m^3^” for the foaming agent denotes the volume of generated foam after 1:60 dilution and foaming. The foam density was approximately 50 kg/m^3^, and the carbon emission factor was estimated per unit volume of generated foam based on relevant literature. Note 2: Cement, wood fiber, fly ash, aggregate, additive, and ecological fertilizer were sourced from local processing plants in Sichuan Province, China, with an average transportation distance of about 30 km. The planting soil was red-bed clay from the Sichuan Basin, with a transportation distance of about 35 km. Mixing water was obtained locally on site and was excluded from the transportation stage. The foaming agent was supplied by Shandong Yousuo Chemical Technology Co., Ltd. (Shandong, China), with a transportation distance of about 1400 km.

**Table 3:** Carbon Sequestration Factors for Plant.

Item	Average Annual CO_2_ Sequestration (kgCO_2_eq/m^2^·a)	References
Vegetation	0.932	Xiao et al. [32]

Note: The annual vegetation carbon sequestration factor of 0.932 kgCO_2_eq/(m^2^·a) in Table 3 was adopted from Reference [32], where it was originally used to represent the average carbon sequestration capacity of green vegetation composed of arbor-shrub and herbaceous layers. Given the current lack of field-measured species-specific carbon sequestration factors that fully match the vegetation configuration considered in this study, this value was used as a reference for vegetation carbon sequestration accounting.

**Table 4:** Results of the orthogonal experiment.

Group	Shear Strength (kPa)	SD	Carbon Emission (kgCO_2_eq)
M1	158	1.00	31.14
M2	168	1.00	32.08
M3	151	1.73	33.02
M4	141	1.73	33.96
M5	170	2.00	46.84
M6	157	1.73	47.46
M7	163	1.73	48.60
M8	165	1.73	49.22
M9	168	1.00	62.50
M10	168	1.00	63.39
M11	168	1.00	63.63
M12	180	2.00	64.51
M13	173	1.73	78.09
M14	188	1.00	78.65
M15	182	1.73	79.33
M16	171	1.00	79.89

**Table 5:** Range Analysis Table.

Evaluation Indicator	Cement	Wood Fiber	Fly Ash	Foaming Agent	Order of Influence
Carbon emission (kgCO_2eq_)	46.44	2.25	0.48	0.09	Cement > Wood fiber > Fly ash > Foaming agent
Shear strength (kPa)	24.00	6.00	13.75	10.25	Cement > Fly ash > Foaming agent > Wood fiber

**Table 6:** Mean estimates and 95% confidence intervals of carbon emissions during the material production stage based on 10,000 Monte Carlo simulations.

Group	Mean(kgCO_2_eq)	95% Confidence Interval (kgCO_2_eq)
M2	32.08	[32.03, 32.13]
M6	47.46	[47.39, 47.53]
M10	63.39	[63.29, 63.50]
M14	78.65	[78.51, 78.79]

## Data Availability

The original contributions presented in this study are included in the article/Supplementary Materials. Further inquiries can be directed to the corresponding author.

## Supplementary Materials

The following supporting information can be downloaded at: https://www.mdpi.com/article/10.3390/ma19112237/s1, Table S1: Carbon emission contributions of individual raw materials during the material production stage for different mix proportions (kgCO_2_eq); Table S2: Itemized efficacy coefficients and comprehensive efficacy coefficients for different mix proportions.
