# Supplementary material for: Net Carbon Sink Potential of Porous Vegetated Concrete: A Life-Cycle Assessment
Source: Materials (Basel). 2026 May 25;19(11):2237. doi: 10.3390/ma19112237 (PMC13258616; doi:10.3390/ma19112237)
Supplement: Supplementary file 1 [file materials-19-02237-s001.zip › materials-4293299-supplementary.pdf]

Table S1. Carbon emission contributions of individual raw materials during the material production stage for different mix proportions (kgCO_{2eq}).

Group	Cement	Wood Fiber	Fly Ash	Foaming Agent	Planting Soil	Aggregate	Additive	Ecological Fertilizer	Water
M1	23.22	2.25	0.16	0.13	0.75	3.10	0.36	1.11	0.05
M2	23.22	3.00	0.32	0.16	0.75	3.10	0.36	1.11	0.05
M3	23.22	3.75	0.48	0.19	0.75	3.10	0.36	1.11	0.05
M4	23.22	4.50	0.64	0.22	0.75	3.10	0.36	1.11	0.05
M5	38.70	2.25	0.32	0.19	0.75	3.10	0.36	1.11	0.05
M6	38.70	3.00	0.16	0.22	0.75	3.10	0.36	1.11	0.05
M7	38.70	3.75	0.64	0.13	0.75	3.10	0.36	1.11	0.05
M8	38.70	4.50	0.48	0.16	0.75	3.10	0.36	1.11	0.05
M9	54.18	2.25	0.48	0.22	0.75	3.10	0.36	1.11	0.05
M10	54.18	3.00	0.64	0.19	0.75	3.10	0.36	1.11	0.05
M11	54.18	3.75	0.16	0.16	0.75	3.10	0.36	1.11	0.05
M12	54.18	4.50	0.32	0.13	0.75	3.10	0.36	1.11	0.05
M13	69.66	2.25	0.64	0.16	0.75	3.10	0.36	1.11	0.05
M14	69.66	3.00	0.48	0.13	0.75	3.10	0.36	1.11	0.05
M15	69.66	3.75	0.32	0.22	0.75	3.10	0.36	1.11	0.05
M16	69.66	4.50	0.16	0.19	0.75	3.10	0.36	1.11	0.05

Table S2. Itemized efficacy coefficients and comprehensive efficacy coefficients for different mix proportions.

Group	Shear Strength Efficacy Coefficient	Carbon Emission Efficacy Coefficient	Comprehensive Efficacy Coefficient
M1	0.84	1	1
M2	0.894	0.971	0.971
M3	0.803	0.943	0.943
M4	0.75	0.917	0.917
M5	0.904	0.665	0.665
M6	0.835	0.656	0.656
M7	0.867	0.641	0.641
M8	0.878	0.633	0.633
M9	0.894	0.498	0.498
M10	0.894	0.491	0.491
M11	0.894	0.489	0.489
M12	0.957	0.483	0.483
M13	0.92	0.399	0.399
M14	1	0.396	0.396
M15	0.968	0.393	0.393
M16	0.91	0.39	0.39
